# The optimization of a rapid low-cost alternative of large-scale medium sterilization

**DOI:** 10.1186/1753-6561-7-S6-P45

**Published:** 2013-12-04

**Authors:** Dominique T Monteil, Cédric A Bürki, Lucia Baldi, David L Hacker, Maria de Jesus, Florian M Wurm

**Affiliations:** 1 Laboratory of Cellular Biotechnology, Faculty of Life Sciences, Ecole Polytechnique Fédérale de Lausanne, 1015 Lausanne, Switzerland; 2 ExcellGene SA, 1870 Monthey, Switzerland

## Background

One of the most important unit operations in upstream animal cell bioprocesses at scales over 100 L is the preparation and sterilization of the medium. This complex, sensitive, and expensive process requires a considerable investment in both material and time [1]. Traditionally, large-scale medium sterilization is performed with costly single-use dead-end filters. To optimize and reduce the cost of this unit operation, we investigated the sterilization of mammalian cell culture medium at volumes larger than 100 L.

## Materials and methods

In this study, an optimization of the cost and time for the sterilization of cell culture medium at volumes larger than 100 L was investigated. Pressure-volume diagrams were completed for both a positive displacement pump (Watson-Marlow 620, Cornwall, England) and a bearingless centrifugal pump (Levitronix PuraLev 600 MU, Zurich, Switzerland) to determined optimal pumping speeds and pressures. The study was completed using 0.25" ID tubing with a gate valve downstream of the pump. The pressure (SciLog SciPres, Madison, WI, USA) and flow rate (Equflow flowsensor, Ravenstein, Netherlands) were measured at diffeFinarent closures of the valve. Independently, a range of different size glass microfiber (GF) pre-filters were tested in combination with and without the dead-end filters by measuring the turbidity (TN100, Eutech Instruments, Singapore). A range of different 0.2 μm dead-end membrane filter materials including polyethersulfone (PES), polyvinylidene fluoride (PVDF), and mixed cellulose ester (ME) were tested using a positive displacement pump. In addition, tangential flow filtration (TFF) was examined with both PES and ME 0.2 μm membranes in comparison to the dead-end filters. A mammalian cell culture medium was filter sterilized at a starting pressure of 500 mbar. The pressure and flow rate were recorded during the filtration until the transmembrane pressure increased to 1200 mbar. The filtration was then stopped at the pressure limit of the tubing connections. Specific filtered medium volume, filter liquid flux rate, and filtrate turbidity were determined for each membrane type.

## Results

**Figure 1:**
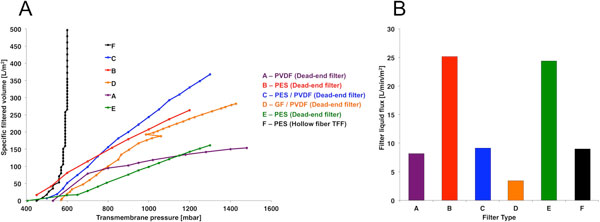
**The calculated specific filtered volume displayed over the changing transmembrane pressure for a range of different filter types (A)**. The calculated filter liquid flux rate for different filter types (B). The filter pore sizes were as followed: A - PVDF 0.45/0.22 μm, B - PES 0.2 μm, C - PES/PVDF 0.2/0.1 μm, D - GF/PVDF 0.5/0.2 μm, E - PES 0.8/0.2 μm, and F - PES 0.2 μm.

The pressure-volume diagram displayed a higher flow rate for the bearingless centrifugal pump (6 to 7 Lpm) in comparison to the peristaltic pump (2.5 Lpm) at the desired pressure of 1000 mbar (data not shown). The turbidity for unfiltered, pre-filtered, and filtered medium was 2.5, 0.75, and 0.2 NTU, respectively, demonstrating the possible benefits of using a pre-filter (data not shown). The filter liquid flux rates ranged from 3 to 25 L/min/m^2 ^for the range of different filters. The PES hollow fiber TFF filters (Spectrum Labs, Breda, Netherlands) displayed a flux rate of 10 L/min/m^2 ^(Figure 1B). The specific filtered volume for the dead-end filters was up to 300 L per m^2 ^of filter surface, while the TFF filter was able to achieve over 1000 L of sterilely filtered medium per m^2 ^of filter surface (Figure 1A).

## Conclusions

The optimization of pumps for the sterile filtration of mammalian cell culture was completed. Our results indicate that a bearingless centrifugal pump could provide twice the flow rate at the desired filtration pressure in comparison to a peristaltic pump. In addition, the bearingless centrifugal pump was able to provide a constant flow in comparison to the peristaltic pump. Pre-filters were found to clarify the medium and thus could further reduce the cost of the filtration. The PES hollow fiber TFF filter was able to filter over three times the sterile medium volume in comparison to the dead-end filters. The TFF filters displayed a similar range of filter liquid flux rates in comparison to the different filters types. This study showed that a hollow fiber TFF coupled with the use of a bearingless centrifugal pump provides a low-cost technology for the rapid large-scale 0.2 μm sterilization of mammalian cell culture medium.
